# Supplementary material for: Causal interplay between lactose intolerance and gut microbiota: a combined bidirectional Mendelian randomization and in vivo validation study
Source: Front Nutr. 2026 Jun 1;13:1803337. doi: 10.3389/fnut.2026.1803337 (PMC13265576; doi:10.3389/fnut.2026.1803337)
Supplement: Supplementary file 1 [file Data_Sheet_1.zip › supplementary materials/Forward/scatter plot/ebi-a-GCST90027815.finngen_R12_E4_LACTONAS.pdf]

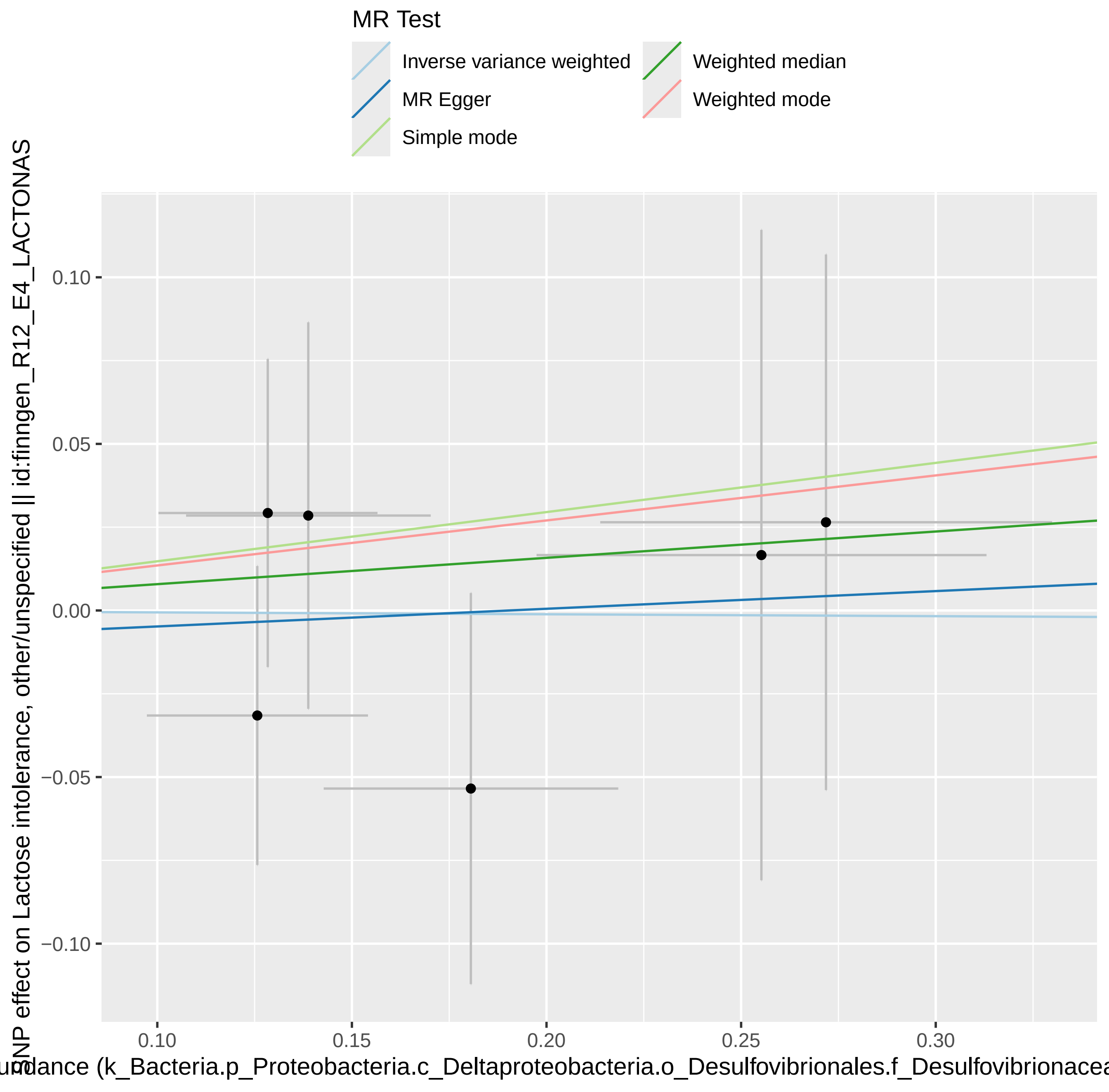
MR Test
Inverse variance weighted
MR Egger
Simple mode
Weighted median
Weighted mode
0.10
0.05
0.00
−0.05
−0.10
0.10
0.15
0.20
0.25
0.30
SNP effect on Lactose intolerance, other/unspecified || id:finngen_R12_E4_LACTONAS
undance (k_Bacteria.p_Proteobacteria.c_Deltaproteobacteria.o_Desulfovibrionales.f_Desulfovibrionacea
